# Long-term *in situ* Eulerian Sea surface temperature records along the Portuguese Coast

**DOI:** 10.1016/j.dib.2024.110287

**Published:** 2024-03-01

**Authors:** Nuno Pessanha Santos, Ricardo Moura, Catarina Santos da Silva, Luisa Lamas, Victor Lobo, Miguel de Castro Neto

**Affiliations:** a Portuguese Military Research Center (CINAMIL), Portuguese Military Academy (Academia Militar), Lisbon 1169-203, Portugal; b Institute for Systems and Robotics (ISR), Instituto Superior Técnico (IST), Lisbon 1049-001, Portugal; c Portuguese Navy Research Center (CINAV), Portuguese Naval Academy (Escola Naval), Almada 2810-001, Portugal; d Centro de Matemática e Aplicações (Nova Math), Universidade Nova de Lisboa, Caparica 2829-516, Portugal; e Portuguese Hydrographic Institute (Instituto Hidrográfico), Rua das Trinas 49, Lisbon 1249-093, Portugal; f NOVA Information Management School (Nova IMS), Universidade Nova de Lisboa, Lisbon 1070-312, Portugal

**Keywords:** Ocean surface temperature, Buoys, Temperature control, Temperature monitoring, Upwelling

## Abstract

Monitoring ocean surface temperature is critical to infer the variability of the upper layers of the ocean, from short temporal scales to climatic change scales. Analysis of the climatological trends and anomalies is fundamental to comprehend the long-term effects of climate change on marine ecosystems and coastal regions. The original data for the dataset presented was collected by the Portuguese Hydrographic Institute (*Instituto Hidrográfico*) using seven Ondograph and Meteo-oceanography buoys anchored offshore along the Portuguese coast to acquire ocean surface temperatures. The original raw data was pre-processed to provide averages over 3-hour periods and daily averages, and this *cleaned* data constitutes the provided dataset. The 3-hour temperature averages were obtained mainly between 2011 and 2015, and the daily temperature averages were obtained in intervals that vary with the considered buoy, having an average interval of 14 years per buoy. The data gathered provides a considerable temporal window, enabling the creation of data series and the implementation of data mining algorithms to develop decision support systems. Collecting data *in situ* makes it possible to validate simulated results obtained using approximation models. This allows for more accurate temperature readings and facilitates testing and correcting created models.

Specifications Table

**Table:** 

Subject	Oceanography
Specific subject area	Spatio-temporal variability of sea surface temperature on the Portuguese coast.
Data format	Processed data in .csv (separate files for each buoy).
Type of data	.csv files (dataset with labels and numbers).
Data collection	Data was collected using seven buoys, three of which were Ondograph (*Datawell Waverider MkIII*), and the remaining four were Meteo-oceanographic (*Oceanor Wavescan*) buoys. The data was collected through temperature sensors on the buoys to obtain 3-hour and daily temperature averages. The 3-hour temperature averages were obtained mainly between 2011 and 2015, and the daily temperature averages were obtained in intervals that vary with the considered buoy, having an average interval of 14 years per buoy. • Buoy 1 | Name: *Leixões* | Time period of daily averages: 1998 – 2019 | Time period of the 3-hour averages: 2011 – 2015 • Buoy 2 | Name: *Raia* | Time period of daily averages: 2010 – 2019 | Time period of the 3-hour averages: 2011 – 2015 • Buoy 3 | Name: *Monican02* | Time period of daily averages: 2010 – 2019 | Time period of the 3-hour averages: 2011 – 2015 • Buoy 4 | Name: *Monican01* | Time period of daily averages: 2009 – 2017 | Time period of the 3-hour averages: 2011 – 2015 • Buoy 5 | Name: *Sines* | Time period of daily averages: 1996 – 2019 | Time period of the 3-hour averages: 2011 – 2015 • Buoy 6 | Name: *Faro* | Time period of daily averages: 1986 – 1992 & 2000 – 2019 | Time period of the 3-hour averages: 2011 – 2015 • Buoy 7 | Name: *FarOff* | Time period of daily averages: 2014 – 2019 | Time period of the 3-hour averages: 2014 – 2015
Data source location	• Buoy 1 | Name: *Leixões* | Coordinates (WGS 84): 41°19.00′N & 008°59.00′W • Buoy 2 | Name: *Raia* | Coordinates (WGS 84): 41°08.9′N & 009°34.9′W • Buoy 3 | Name: *Monican02* | Coordinates (WGS 84): 39°33.6′N & 009°12.6′W • Buoy 4 | Name: *Monican01* | Coordinates (WGS 84): 39°30.94′N & 009°38.24′W • Buoy 5 | Name: *Sines* | Coordinates (WGS 84): 37°55.3′N & 008°55.7′W • Buoy 6 | Name: *Faro* | Coordinates (WGS 84): 36°54.3′N & 007°53.9′W • Buoy 7 | Name: *FarOff* | Coordinates (WGS 84): 36°23.90′N & 008°04.10′W
Data accessibility	Repository name: *Figshare* All data can be accessed at the following link: https://dx.doi.org/10.6084/m9.figshare.25024592 The archive content can be publicly accessed and downloaded without needing any registration.

## 1 Value of the Data

• These data are useful for understanding the Spatio-temporal variability of the sea surface temperature along the Portuguese coast.
• The data were gathered *in situ* using Ondograph and Meteo-oceanographic buoys, which enabled the data to be captured in real-time and over large periods.
• The analysis of the data can be useful for the climatological characterization of the sea surface temperature, considering the seasonality and durability of coastal upwelling events along the Portuguese coast.
• The data can be used to validate numerical or predictive models, providing ground truth for characterizing the models’ errors.
• The data is useful for understanding long-term climate change patterns and trends along the Portuguese coast.
• Oceanography researchers and scientists can benefit from real data gathered from buoys in seven different locations along the Portuguese coast, with daily temperature averages having an average interval of 14 years per buoy, enabling the creation of data series and the implementation of data mining algorithms.

## 2 Background

The data was collected over the years along the Portuguese coast using Ondograph (*Datawell Waverider MkIII*) and Meteo-oceanographic (*Oceanor Wavescan*) buoys. The fundamental mission of the Portuguese Hydrographic Institute (*Instituto Hidrográfico*) is to carry out activities related to the sciences and techniques of the sea [1]. Its aim is to apply these activities in the military area and contribute to the country's scientific and maritime development, namely in protecting the environment. One of the many tasks performed along the Portuguese coast is measuring the spatio-temporal variability of the sea surface temperature.

## 3 Data Description

**Fig. 1:**
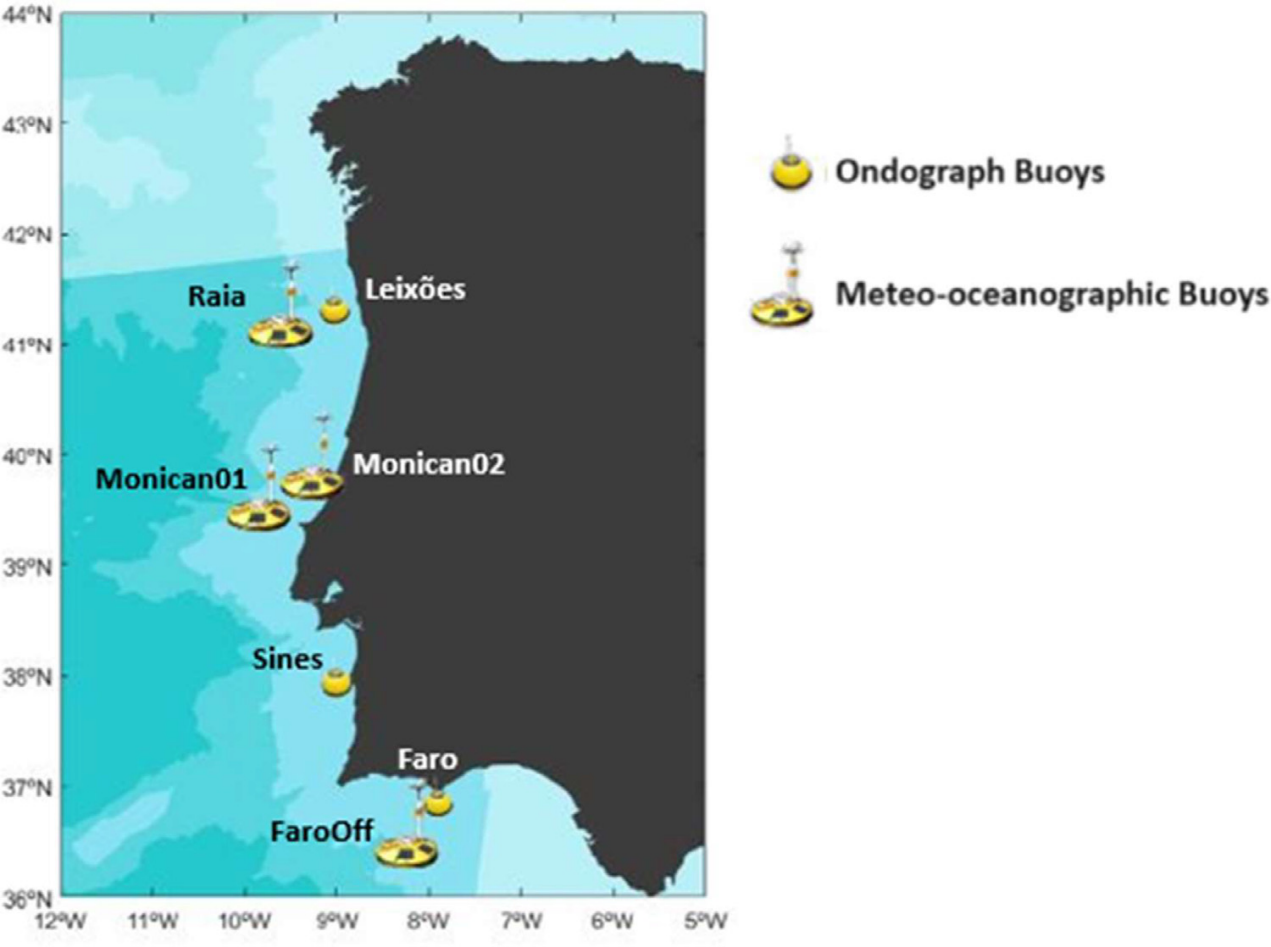
Location of the study area and the location of the respective buoys.

The dataset contains temperature registers [2] collected from sensors installed on seven buoys, which can perform Sea Surface Temperature (SST) monitoring of the Portuguese coastal ocean [3,4], as illustrated in Fig. 1. Three Ondograph (*Datawell Waverider MkIII*) buoys were used for the data acquisition, and the remaining four were Meteo-oceanographic (*Oceanor Wavescan*) buoys. The information related to each buoy is described in Table 1.

**Table 1:** Summary of the collected data information per Buoy.

Buoy Number	Name	Time period of the daily averages	Time period of the 3-hour averages
1	*Leixões*	1998 – 2019	2011 – 2015
2	*Raia*	2010 – 2019	2011 – 2015
3	*Monican02*	2010 – 2019	2011 – 2015
4	*Monican01*	2009 – 2017	2011 – 2015
5	*Sines*	1996 – 2019	2011 – 2015
6	*Faro*	1986 – 1992	2011 – 2015
		2000 – 2019	
7	*FarOff*	2014 – 2019	2014 – 2015

Each buoy has two different “.xlsx” files in the dataset, accounting for fourteen files. They are named "Buoy_*Number*_*Name*_A.xlsx" and "Buoy_*Number*_*Name*_B.xlsx". The "Buoy_*Number*_*Name*_A.xlsx" file contains information about the ocean surface temperature collected every three hours, while the "Buoy_*Number*_*Name*_B.xlsx" file includes information on the daily average ocean temperature records. The fields available in each file are presented in Table 2 and Table 3. All data were collected directly from the buoy's sensors and then averaged over the time periods considered. Newly created variables are available to allow the calculation of weighted average temperatures.

**Table 2:** "Buoy_*Number*_*Name*_A.xlsx" content description.

Variable	Description
*Inter_3h*	Description of the considered 3-hour interval (YYYY-MM-DD___HH _HH_h)
*Date*	Datalogging date (YYYY-MM-DD)
*inter*	Logging Interval (HH _HH_h)
*SST_inter*	Sea surface temperature 3-hour average in degrees Celsius
*Year*	Year number
*MonthNumber*	Month number

**Table 3:** "Buoy_*Number*_*Name*_B.xlsx" content description.

Variable	Description
*Date*	Datalogging date (YYYY-MM-DD)
*SST_Day*	Sea surface temperature daily average in degrees Celsius
*Year*	Year number
*MonthNumber*	Month number
*Weight*	A day weighs one unless there are no temperature records, in which case the weight is zero. The calculation is based on the number of 3-hour temperature intervals with records divided by eight (eight three-hour intervals in 24 h)

**Fig. 2:**
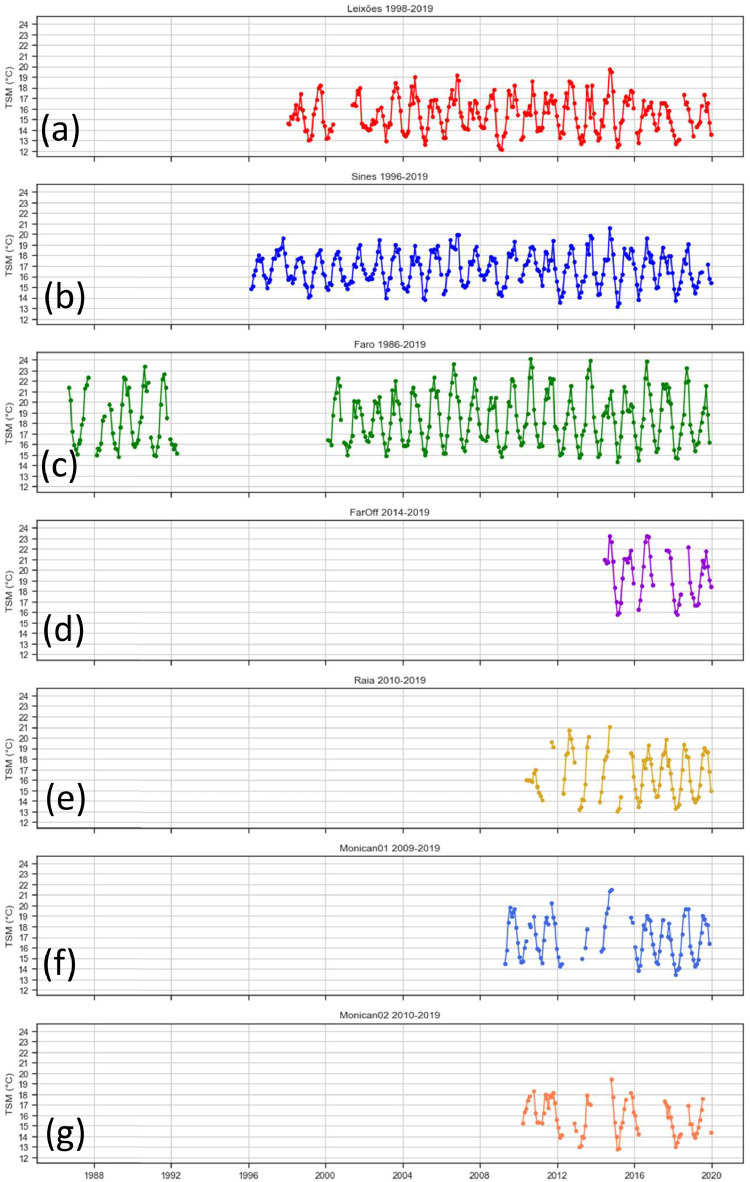
Monthly averages of the recorded sea surface temperatures. The represented buoy plots are (a) *Leixões*, (b) *Sines*, (c) *Faro*, (d) *FarOff*, (e) *Raia*, (f) *Monican01*, and (g) *Monican02.*

Through the analysis of the data presented in Table 1, it is possible to state that the data was not acquired uniformly over a specific time interval mainly because the buoys were not installed simultaneously and, sometimes, the data acquisition was not successfully performed due to some failure in the acquisition process. Fig. 2 represents the monthly average sea surface temperature records, allowing a clear interpretation and knowledge about the years and months for which we have available information in the provided dataset. The 3-hour averages were mainly obtained between 2011 and 2015 due to the overlap between all buoys data gathering during this time frame, as shown in Fig. 2. The *FarOff* buoy had no data available between 2011 and 2014, so the 3-hour averages were only considered for 2014 and 2015, as described in Table 1. If a different data period and more detailed data are required, it can be requested from the Portuguese Hydrographic Institute's website [1].

## 4 Experimental Design, Materials and Methods

All the data were acquired using seven buoys along the Portuguese coast, as illustrated in Fig. 1. A summary of each buoy location and data acquisition conditions can be seen in Table 4. The Portuguese Hydrographic Institute (*Instituto Hidrográfico*) performed buoy maintenance and data acquisition during all periods [1]. Corrective maintenance is carried out as needed, and preventive maintenance is ideally done every six months to a year. During maintenance, all sensors are calibrated in a certified laboratory belonging to the Portuguese Hydrographic Institute to reduce errors that may arise from long-term usage. This institute is essential for Portugal, a coastal state, and it ensures activities related to marine sciences and techniques that are used for applications in national defense and contribute to the country's development in the scientific and environmental areas.

**Table 4:** Resume of each buoy location and data acquisition conditions.

Buoy Number	Name	Position (WGS 84)	Measure depth (m)	Depth at anchor (m)	Buoy type
1	*Leixões*	41°19.00′N 008°59.00′W	0.7	83	Ondograph
2	*Raia*	41°08.9′N 009°34.9′W	1.0	1600	Meteo-oceanographic
3	*Monican02*	39°33.6′N 009°12.6′W	1.0	90	Meteo-oceanographic
4	*Monican01*	39°30.94′N 009°38.24′W	1.0	2000	Meteo-oceanographic
5	*Sines*	37°55.3′N 008°55.7′W	0.7	97	Ondograph
6	*Faro*	36°54.3′N 007°53.9′W	0.7	93	Ondograph
7	*FarOff*	36°23.90′N 008°04.10′W	1.0	1334	Meteo-oceanographic

**Fig. 3:**
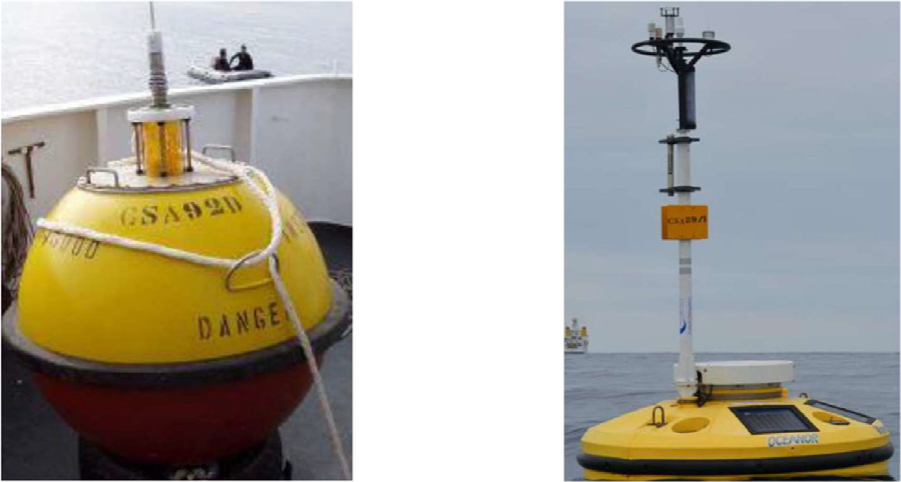
Used Byous: *Datawell Waverider MkIII* (*left*) and *Oceanor Wavescan* (*right*).

The *Datawell Waverider MkIII* ondograph buoys (Fig. 3) that were used have a temperature resolution of 0.05 °C and an accuracy level of less than 0.1 °C [5,6]. The data collected from these buoys was transmitted using a Very High-frequency (VHF) data link. The Meteo-oceanographic buoys used were Oceanor Wavescan, as shown in Fig. 3. These buoy sensors have a temperature resolution of 0.001 °C and an accuracy of 0.03 °C for water temperature measurement. The collected data from these buoys was transmitted using a satellite link [7,8]. A more detailed technical description of the used buoys can be consulted or requested directly from the manufacturer's website [5,8]. The gathered data resolution and accuracy are very high, making it compatible with most desired applications and studies. The Meteo-oceanographic buoys gather highly reliable data that undergoes an additional data validation process to ensure its accuracy. In addition, the obtained data undergoes quality control based on the norms of the European Global Ocean Observing System (EuroGOOS) [9]. This process allows for calculating a metric that guarantees the quality of the data. The validated measurement number and percentage are described in Table 5.

**Table 5:** Relation between the collected data dimension and the obtained accurate measurements.

Buoy Number	Name	Position (WGS 84)	Sample Size	Accurate data (%)	Buoy type
2	*Raia*	41°08.9′N 009°34.9′W	59,414	95.3 %	Meteo-oceanographic
3	*Monican02*	39°33.6′N 009°12.6′W	62,306	87.2 %	Meteo-oceanographic
4	*Monican01*	39°30.94′N 009°38.24′W	44,691	99.6 %	Meteo-oceanographic
7	*FarOff*	36°23.90′N 008°04.10′W	35,923	99.3 %	Meteo-oceanographic

## Limitations

Our ability to collect *in-situ* measurements is limited to only seven specific locations along the vast Portuguese coastline due to our use of a restricted buoy network. Although our dataset is valuable, it is limited in scope by monitoring infrastructure constraints.

## Ethics Statement

The authors confirm that the current work does not involve human subjects, animal experiments, or data collected from social media platforms, complying with all ethical requirements for publication in Data in Brief.

## CRediT authorship contribution statement

**Nuno Pessanha Santos:** Conceptualization, Methodology, Validation, Formal analysis, Investigation, Data curation, Writing – original draft, Writing – review & editing, Visualization, Supervision, Project administration, Funding acquisition. **Ricardo Moura:** Conceptualization, Methodology, Software, Validation, Formal analysis, Investigation, Data curation, Writing – review & editing, Visualization, Supervision, Project administration, Funding acquisition. **Catarina Santos da Silva:** Methodology, Software, Validation, Formal analysis, Investigation, Data curation, Visualization. **Luisa Lamas:** Validation, Formal analysis, Data curation. **Victor Lobo:** Conceptualization, Validation, Writing – review & editing, Project administration, Funding acquisition. **Miguel de Castro Neto:** Conceptualization, Project administration, Funding acquisition.

## Data Availability

Long-term In Situ Eulerian Sea Surface Temperature Records along the Portuguese Coast (Original data) (Figshare).
